# A computational model of induced pluripotent stem-cell derived cardiomyocytes for high throughput risk stratification of KCNQ1 genetic variants

**DOI:** 10.1371/journal.pcbi.1008109

**Published:** 2020-08-14

**Authors:** Divya C. Kernik, Pei-Chi Yang, Junko Kurokawa, Joseph C. Wu, Colleen E. Clancy

**Affiliations:** 1 Department of Physiology and Membrane Biology, Department of Pharmacology, School of Medicine, University of California, Davis, Davis, California, United States of America; 2 Department of Bio-Informational Pharmacology, School of Pharmaceutical Sciences, University of Shizuoka, Shizuoka, Japan; 3 Stanford Cardiovascular Institute, Department of Medicine, Division of Cardiovascular Medicine, Stanford University School of Medicine, Stanford, California, United States of America; King's College London, UNITED KINGDOM

## Abstract

In the last decade, there has been tremendous progress in identifying genetic anomalies linked to clinical disease. New experimental platforms have connected genetic variants to mechanisms underlying disruption of cellular and organ behavior and the emergence of proarrhythmic cardiac phenotypes. The development of induced pluripotent stem cell-derived cardiomyocytes (iPSC-CMs) signifies an important advance in the study of genetic disease in a patient-specific context. However, considerable limitations of iPSC-CM technologies have not been addressed: 1) phenotypic variability in apparently identical genotype perturbations, 2) low-throughput electrophysiological measurements, and 3) an immature phenotype which may impact translation to adult cardiac response. We have developed a computational approach intended to address these problems. We applied our recent iPSC-CM computational model to predict the proarrhythmic risk of 40 KCNQ1 genetic variants. An I_Ks_ computational model was fit to experimental data for each mutation, and the impact of each mutation was simulated in a population of iPSC-CM models. Using a test set of 15 KCNQ1 mutations with known clinical long QT phenotypes, we developed a method to stratify the effects of KCNQ1 mutations based on proarrhythmic markers. We utilized this method to predict the severity of the remaining 25 KCNQ1 mutations with unknown clinical significance. Tremendous phenotypic variability was observed in the iPSC-CM model population following mutant perturbations. A key novelty is our reporting of the impact of individual KCNQ1 mutant models on adult ventricular cardiomyocyte electrophysiology, allowing for prediction of mutant impact across the continuum of aging. This serves as a first step toward translating predicted response in the iPSC-CM model to predicted response of the adult ventricular myocyte given the same genetic mutation. As a whole, this study presents a new computational framework that serves as a high throughput method to evaluate risk of genetic mutations based-on proarrhythmic behavior in phenotypically variable populations.

## Author summary

In the last decade, there has been tremendous progress in identifying genetic mutations linked to clinical diseases, such as cardiac arrhythmia. Many experimental platforms have been developed to study this link, including induced pluripotent stem cell-derived cardiomyocytes (iPSC-CMs). IPSC-CMs are patient-derived cardiac cells which allow for the study of genetic variants within a patient-specific context. However, experimentally iPSC-CMs have certain limitations, including: (1) they exhibit variability in behavior within cells that are apparently genetically identical, and (2) they are immature compared to adult cardiac cells. In our study, we have developed a computational approach to model 40 genetic variants in the KCNQ1 gene and predict the proarrhythmic risk of each variant. To do this, we modeled the ionic current determined by KCNQ1, I_Ks_, to fit experimental data for each mutation. We then simulated the impact of each mutation in a population of iPSC-CMs, incorporating variability across the population. We also simulated each variant in an adult cardiac cell model, providing a link between iPSC-CM response to mutants and adult cardiac cell response to the same mutants. Overall, this study provides a new computational framework to evaluate risk of genetic mutations based-on proarrhythmic behavior diverse populations of iPSC-CM models.

## Introduction

The impact of genetic variation on cardiac electrical activity is increasingly understood through identification and characterization of genetic anomalies in cardiac ion channel encoding genes, and their causal relationship to patient phenotype [1–3]. Understanding how variation in cardiac genes impacts cardiac function is important for treating and understanding complex genetic and inherited disorders, distinguishing between benign and hazardous variants of unknown significance (VUS), and revealing differential responses to drug interventions [4, 5]. For example, mutations in the KCNQ1 gene have been linked to cardiac repolarization abnormalities, including long QT syndrome (LQTS) [6], although the impact of specific mutations is often unknown. Genetic defects in KCNQ1 linked to LQTS, known as LQT1, cause a decrease in the slow delayed rectifier potassium current (I_Ks_), resulting in prolongation of the action potential at the cellular level and clinical prolongation of the QT interval [7].

Induced pluripotent stem-cell derived cardiomyocytes (iPSC-CMs) have been utilized as a novel *in vitro* tool to reveal insights into patient-specific disease mechanisms [8–10]. iPSC-CMs constitute a powerful approach because they are patient-derived cells that retain the genetic information of the donor patients or cell line and can show patient-specific genotype-phenotype relationships, including genetic disease phenotypes such as LQT1 [11–16]. iPSC-CMs also have unique potential to provide a human physiological context to evaluate the impact of a genetic mutation in an *in vitro* human cardiac environment [17]. iPSC-CMs have further proven to be a powerful tool in evaluating VUS and linking genetic variants to their clinical outcomes [18, 19]. However, evaluating the true significance of VUS mutations will require more than a patient-specific understanding, as a VUS may result in varied phenotypes both within families and across populations [20, 21]. Currently, evaluation of VUS in iPSC-CMs population studies are limited by the relatively low throughput approach of patch-clamp evaluation of cell-specific response [18, 19, 22]. Understanding how variants differentially impact the diverse range of patient genetics across a population will be critical to understanding the clinical significance and treatment of genetic disorders.

The development of computational models that incorporate parameter variation, as a means to explore all possible population phenotypes, provide a high-throughput way to analyze cardiomyocyte phenotypic variability [23–25]. Population-based modeling can link known effects of genetic mutations in a single patient, or an experimental cell model, to the differential effect of a genetic mutation across a population of patients [26, 27], depending on the collective expression of all cardiac ion channels [28, 29]. Population-based modeling provides a high-throughput method to examine trends across diverse cellular phenotypes, while also allowing for mechanistic insights into individual rare events observed in a particular model.

Vanoye *et al*. recently published a novel dataset of KCNQ1 mutations expressed in Chinese hamster ovary (CHO) cells, characterized using automated planar patch clamp [30]. The functional changes in KCNQ1 for each mutation can be implemented as relative changes in our previously developed iPSC-CM I_Ks_ model. Using a high-throughput population-based computational modeling approach, we analyzed differential mutant impact across diverse whole-cell iPSC-CM phenotypes. We also considered KCNQ1 mutations with known clinical phenotypes and developed a framework to computationally predict the severity of KCNQ1 mutations. Additionally, the matched genetic variant models were incorporated into an adult myocyte *in silico* to predict mutation impact across a continuum of maturation. In the future, the approach can be expanded to any cardiac target, genetic perturbations, pharmacological interventions, and the complex behavior produced by multi-drug or mutant interactions.

## Results

### iPSC-CM Wild-Type Model Population

The recently developed Kernik *et al*. 2019 iPSC-CM population provides an ideal system to analyze cell-to-cell variability in response to genetic mutations within the human physiological system [31]. The iPSC-CM experimental system has been developed for studying genetic perturbations in the true physiological background of a human myocyte [8, 10, 13, 32], and are being increasingly utilized in the study of VUS [18, 19]. Utilizing a computational model of iPSC-CM to study the role of cell-to-cell variability in response to genetic mutations is the best way to ensure an available system for model validation in future studies. The Kernik 2019 population of iPSC-CM models includes experimentally observed variation in kinetic parameters and maximal conductance for five key ionic currents: I_Kr_, I_CaL_, I_Na_, I_K1,_ and I_f_. The resulting population predicts a wider range of whole-cell action potential morphology, which is shown to be predictive of the experimentally observed range of iPSC-CM action potential morphologies [31]. Utilizing this population of iPSC-CM models with kinetic variation allows for the comparison of cell-to-cell variability in response to genetic mutations, including how mutant kinetics manifest differently depending on kinetics of all other ionic currents. Moreover, investigating the same genetic perturbations in adult cardiac myocyte models and comparing to clinical phenotypes, when known, provides additional evidence to support the use of the iPSC-CM model system for study of genetic perturbation.

### Test Set 1: *Mutant Model Optimization*

**Fig 1:**
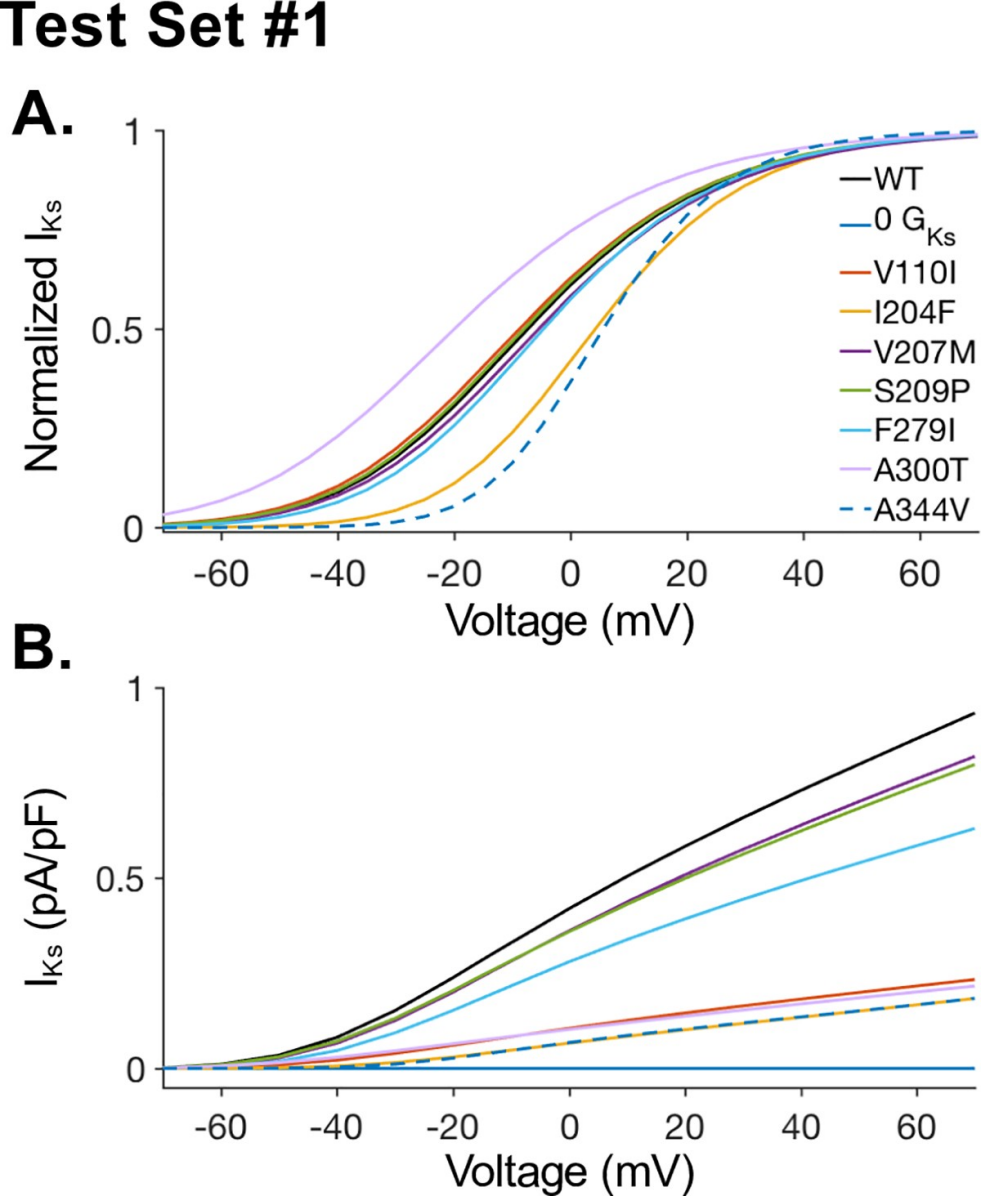
Model optimization to test set 1 data. The iPSC-CM I_Ks_ model was optimized to experimental data for KCNQ1 mutations in test set 1 (TS1). TS1 includes only mutations with known clinical phenotypes. Experimentally observed change relative to the wild-type in Vanoye et al. was used to fit each mutant I_Ks_ model [1]. Models were optimized to data for V_1/2_, k, and current density. **(A)** Steady-state activation curves and **(B)** Current-voltage (I-V) relationships are shown for each mutant model in TS1. Additionally, complete I_Ks_ block (G_Ks_ = 0) was included to represent mutations resulting in insufficient current density to allow measurement. Complete block is not included in panel A, for clarity.

We first compiled a set of KCNQ1 mutations that have been identified in terms of both their effect on the I_Ks_ channel and in terms of patient phenotype. This set of mutations is defined as test set 1 (TS1). To analyze the impact of mutations characterized in TS1, experimental data from Vanoye *et al*. was used to develop a computational model of I_Ks_ that incorporated the kinetic effects of each mutation [30]. For each mutant the I_Ks_ model (Eqs 1–3) were fit to the relative change in G_Ks_, V_1/2_, and k between the wild-type (WT) and mutant KCNQ1. Models were optimized to fit measurements by Vanoye *et al*. recorded using automated patch clamp [30]. The resulting activation curves (x_act,∞_) and I-V curves for the WT model and each TS1 I_Ks_ mutant model are shown in Fig 1A and 1B, respectively. All of the TS1 mutations have known clinical phenotypes in patients, as detailed in the ClinVar database [33]. Based on ClinVar assessments of TS1 mutations, nine mutants are LQTS pathogenic (Y111C, L114P, P197L, C122Y, E160K, R174C, I204F, A344V, V110I), one mutant is LQTS likely pathogenic (A300T), one mutant is short QT pathogenic (F279I), one mutant is atrial fibrillation pathogenic (S209P), and one mutant is likely benign (V207M). Six of the pathogenic mutants in TS1, which result in insufficient current to measure KCNQ1 current density or V_1/2_ (Vanoye et al, supplemental table S4a [30]), were modeled as complete I_Ks_ block (G_Ks_ = 0pA/pF).

### Test Set 1: Predicted *Impact of LQT1 on AP Morphology*

**Fig 2:**
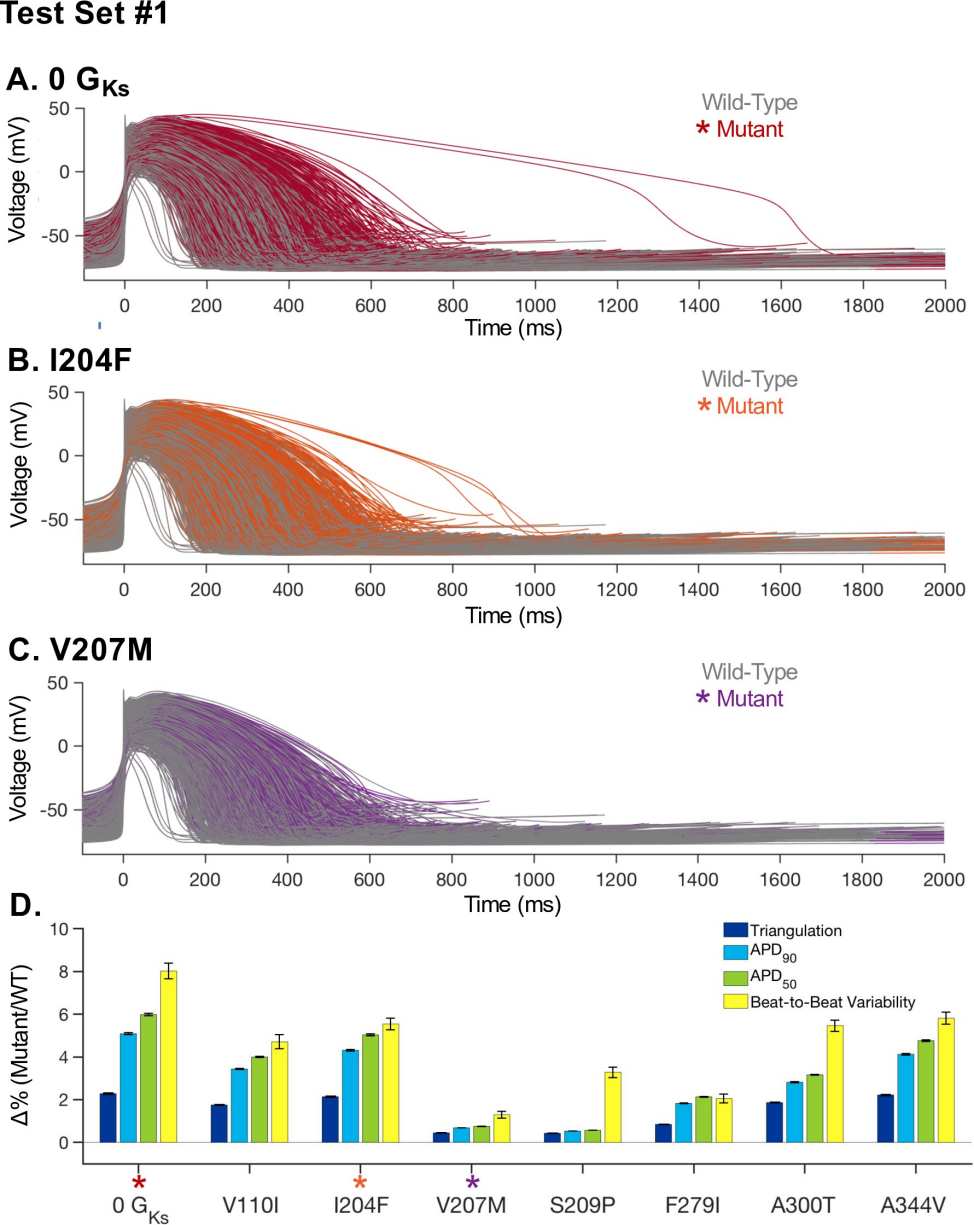
Population-based modeling results for test set 1 mutations in iPSC-CMs computational models. **(A)** For a random subset of the model population (n = 2818), APs are shown for wild-type (grey) and complete I_Ks_ block (0 G_Ks_, red). **(B)** For a random subset of the model population (n = 2826), APs are shown for wild-type (grey) and the I204F mutant (orange). **(C)** A random subset from the model population (n = 2826) are shown for APs for wild-type (grey) and the V207M mutant (purple). **(D)** For each mutant cell in TS1, action potential duration 90% (APD_90_), APD_50_, action potential (AP) triangulation, and APD_90_ beat-to-beat variability were computed relative to the wild-type. AP Triangulation (APD_90_-APD_30_) and beat-to-beat variability (|APD_90_,n-APD_90_,n+1|, for beat n) were averaged over 40 beats with a physiological noise current. The bar graph shows the mutant population average and standard deviation for each output. APs for mutations highlighted with colored stars are shown in panel A-C.

For each mutant in TS1_,_ we replaced the wild-type I_Ks_ model in our control population of iPSC-CM models with the optimized mutant I_Ks_ model. This resulted in a population of iPSC-CM models for each mutation. For a selection of mutations, a subset of APs are shown for the WT and mutant in Fig 2A–2C. It can be seen that some cells within the population are more susceptible to mutations, with several iPSC-CM models showing considerably more prolongation in action potential duration (APD). Total I_Ks_ block and the I204F mutation fall into this category. For each model in each mutant population, AP morphology markers were measured and compared to WT behavior in the same cellular model. The results of this analysis for APD_50_ and APD_90_ are shown in Fig 2D.

The TS1 mutations resulted in a population of 11091–10887 models that were spontaneously beating and fully repolarizing (AP amplitude over 70 mV, resting voltage over 40 mV, and no repolarization abnormalities). Models with repolarization failures did occur and are included in the study as exemplars of pathogenic behavior. The models which developed repolarization failure after implementing each mutation were tracked and reported as a percentage of the total WT population in Table 1. Severe mutants had the highest percentage of models excluded from the population due to repolarization abnormalities.

**Table 1:** Comparison of ClinVar and Computational Results for TS1.

Mutations ordered by size of subpopulation above threshold:				Repolarization Failure (%)
4%	8%	10%		
Y111C*	Y111C*	Y111C*		4.7
L114P*	L114P*	L114P*		4.7
P197L*	P197L*	P197L*		4.7
C122Y*	C122Y*	C122Y*		4.7
G179S*	G179S*	G179S*		4.7
R231C*	R231C*	R231C*		4.7
L236R*	L236R*	L236R*		4.7
R174C*	R174C*	R174C*		4.7
I204F	I204F	I204F		4.4
A344V	A344V	A344V		4.3
V110I	V110I	V110I		4.0
A300T	A300T	A300T		3.8
F279I	F279I	F279I		3.5
V207M	V207M	V207M		3.0
S209P	S209P	S209P		2.9

LQTS

LQTSLikelyPathogenic

Non‐LQTSpathogenic

LikelyBenign

*modeled as GKs = 0A/F

Previous studies have suggested that utilizing only AP prolongation as an indicator of critical change to AP morphology is an inadequate indication of proarrhythmic phenotypes [34, 35]. To further analyze the predicted impact of KCNQ1 mutations on iPSC-CMs, each cell in each mutant population was also simulated for 40 beats with a physiological noise current. The beat to beat variability of APD_90_ (defined in methods) and triangulation of the APs (APD_90_-APD_30_) was analyzed following a simulation with applied noise. The bar graph in Fig 2D shows the result of this simulation for each mutation as a percent change compared to the wild-type population, as well as the change in APD_90_ and APD_50_ compared to the wild-type population.

### TS1: Development of an LQTS Severity Indication

After comparing iPSC-CM AP morphology and temporal responses to perturbation by KCNQ1 mutations, we developed a framework to categorize the predicted severity of response to each mutant. We considered three criteria in developing a severity test to classify each I_Ks_ mutation. We considered the following criteria for each cell in each mutation population:

1. Increase in the triangulation across 40 beats with physiological noise in the mutant compared to the WT.
2. Increase in average beat-to-beat variation across 40 beats with physiological noise in the mutant compared to the WT.
3. Increase in APD_90_ compared to the WT.

**Fig 3:**
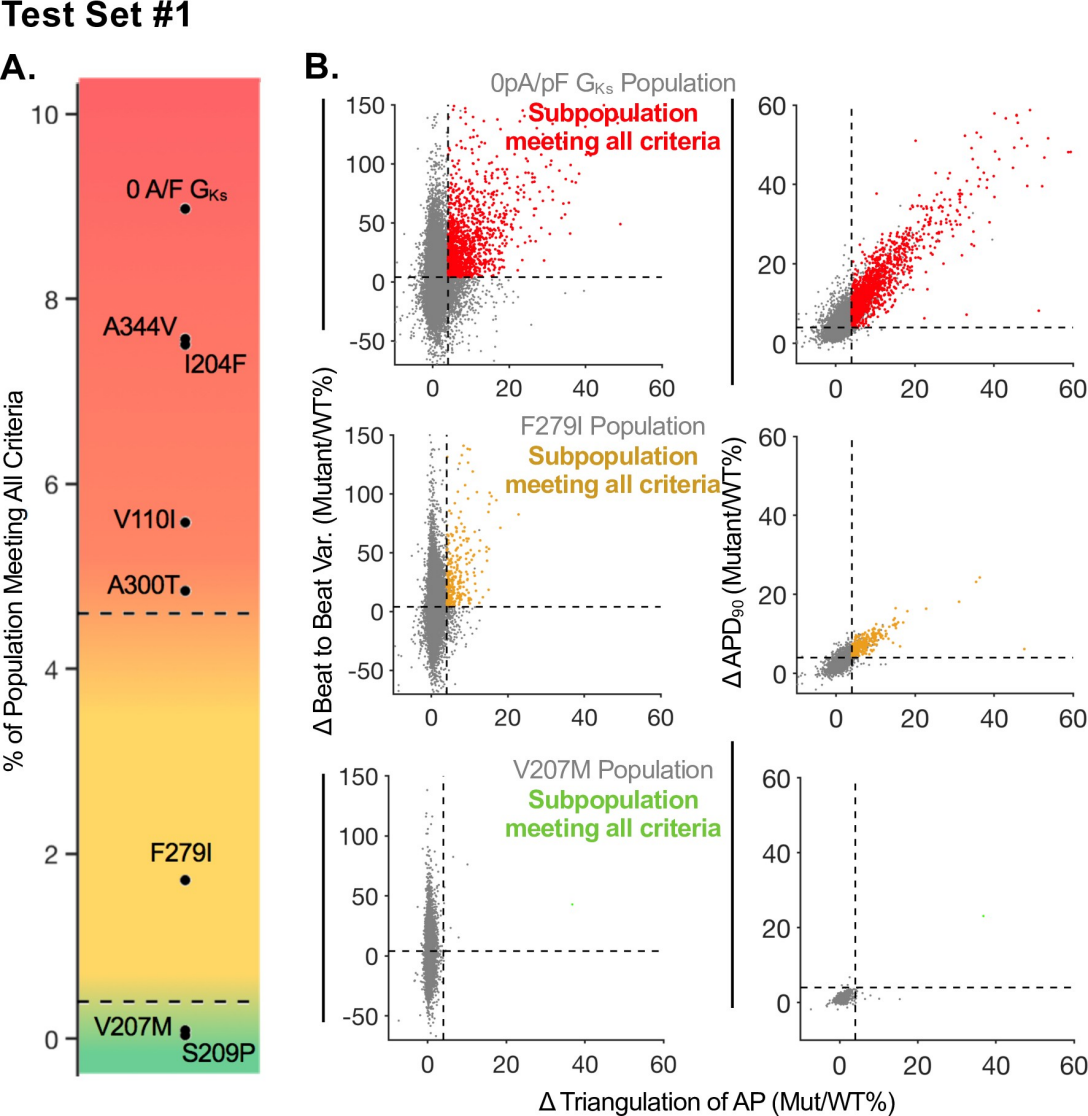
Demonstration of an LQTS severity framework for test set 1. **(A)** Stratification of LQT severity using threshold criteria for APD_90_, Triangulation, and Beat-to-Beat Variability. Red corresponds to the most severe LQTS mutations, green corresponds to benign mutations for LQTS, and yellow highlights the region of severity which cannot be classified utilizing the known clinical phenotypes. **(B)** Three example mutant populations with colors indicating the subpopulation of cells meeting the set criteria. Red highlighted points in the top row represent the 8.97% of model cells in the 0 G_Ks_ population surpassing the 4% threshold for all three criteria. Yellow highlighted points in the middle row represent the 1.71% of model cells in the F279I population surpassing the 4% threshold for all three criteria. Green highlighted points in the bottom row represent the 0.09% of cells in the V207M population surpassing the 4% threshold for all three criteria.

For each mutant population, we considered the percent of cellular models which surpassed a given threshold for the three criteria above. Using this framework with a 4% threshold, we stratified the mutation populations in TS1 to determine the severity of each mutation. The results of this stratification are shown in Fig 3A for TS1. We tested a range of thresholds (4, 5, 8, 10, 15, and 20% change) and saw the same relative stratification of TS1. A comparison of 3 tested thresholds (4%, 8%, and 10%) are shown in Table 1. The table shows the level of clinical of severity (colored in red, yellow, blue and green) based on the ClinVar assertion for each mutation.

In Fig 3B, we show examples of how the criteria and thresholds were applied for example mutant populations. Percent change compared to WT was calculated for each of the three criteria (Fig 3B, left: triangulation and beat-to-beat variation; Fig 3B, right: beat-to-beat variation and APD_90_). All cells in the example mutant population are shown in Fig 3B as individual dots in the scatter plot. The model cells that met all three criteria above the 4% threshold are highlighted in red for the I_KS_ knockout, yellow for the F278I mutation, and green for the V207M mutation. All cells below the threshold criteria are indicated by a grey dot. Comparing the most severe mutant (0 G_Ks_) and a low severity mutation (V207M), there is a much-increased spread in cellular outputs (grey and colored) for the severe mutation. This spread is due to the greater change in AP behavior, compared to WT, for each cell in the severe mutant population. Additionally, there are many more highlighted red points for the severe knockout mutation, than green highlighted points for the predicted low severity mutation (V207M).

The six KCNQ1 mutations which had insufficient current density to optimize the I_Ks_ model (with either ≤0% current density measured experimentally relative to WT, or V_1/2_ could not be determined), have all been classified as pathogenic LQTS mutations in the ClinVar database. Four mutations (I204F, A344V, V110I, and A300T) classified as LQTS mutations (pathogenic or likely pathogenic) had sufficient experimental data to optimize a mutant I_Ks_ model. Three of these mutations have all been observed to prolong patient QT interval and are classified as pathogenic LQTS in the ClinVar database (I204F, A344V, V110I). Additionally, A300T has been observed to prolong the QT interval in a clinical study, but it is either a recessive variant or has incomplete penetrance in patients [36]. These 4 mutant populations, as well as the population with 0 G_Ks_, were predicted to fall into the highest severity stratification in our computational analysis, as shown in Fig 3A.

One mutation included in TS1 is classified as a likely benign variant in the ClinVar database (V207M) and falls in the benign stratification shown in Fig 3A. Finally, there are two mutations which are classified as pathogenic, but for diseases other than LQT (non-LQT pathogenic) as follows: The S209P mutation falls at the lowest severity stratification of our analysis and clinically has been associated with atrial fibrillation (AF). In the clinical study, an AF patient with the S209P mutation, the affected patient had no difference in corrected QT (QTc) interval compared to the unaffected family members [37].

Finally, there was one mutation (F279I) in TS1 which was not clearly categorized by our analysis. Interestingly, there is also some lack of clarity in other experimental studies characterizing this mutation. Clinically, F279I has been classified as a short QT syndrome mutation in the clinical study by Moreno *et al*., where they also examined the impact of the mutation on current density in COS7 cells with mutant vs. WT KCNQ1 and observed a gain of current density [38]. This is the opposite effect for F279I mutant KCNQ1 current density observed in the Vanoye *et al*. data used to optimize our mutant model. The Vanoye *et al*. data showed a 32% reduction in current density with the F279I mutation. Our framework predict that F279I falls in the range that cannot be clearly classified as pathogenic or benign. This range is highlighted in yellow, between the dashed lines, in Fig 3A.

Based on the 4% threshold and the ClinVar assertions, we determined “cut-offs” for pathogenic and benign mutants, represented as the dashed lines in Fig 3A. The cut-off for pathogenic mutants was set for mutants with more than 4.6% of model cells surpassing the above three criteria (top dashed line, Fig 3A). The cut-off for benign mutants was set for mutants with less than 0.25% of model cells surpassing the above three criteria (lower dashed line, Fig 3A).

### Test Set 2: *Comparison of phenotypic variability within mutation populations*

**Fig 4:**
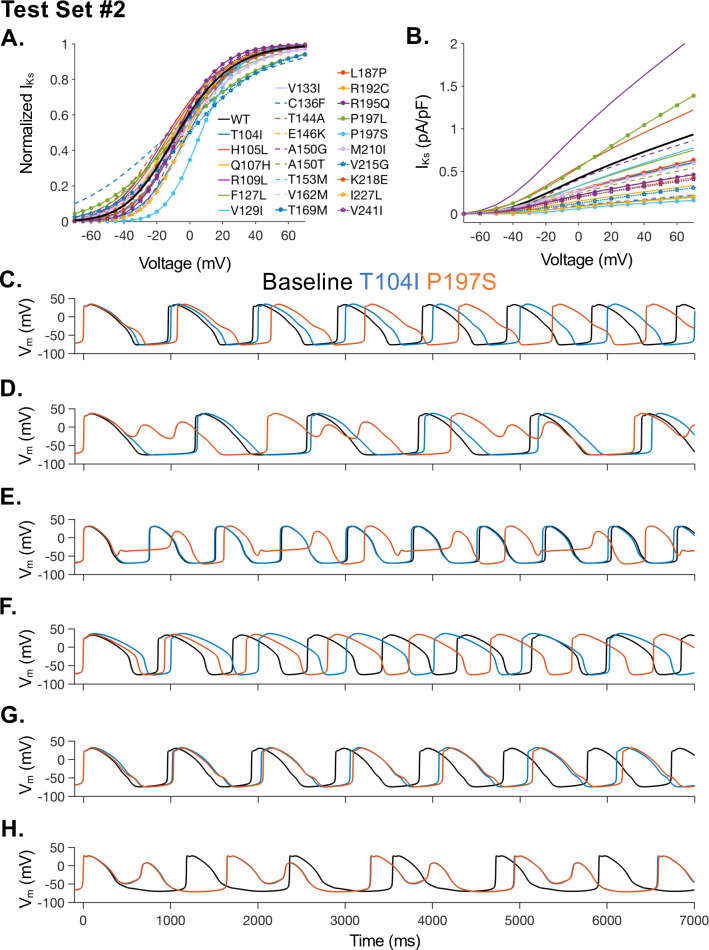
Model optimization for test set 2 (TS2) and sample APs comparing selected mutations in TS2. **(A)** Steady-states activation and **(B)** IV relationships resulting from optimization of the iPSC-CM I_Ks_ model to fit experimental results for TS2 mutations. **(C-H)** Wild-Type (black), T104I mutant (blue), and P197S mutant APs are shown for several sample iPSC-CM models within our population. Mutations were selected to demonstrate the greatest average APD_90_ prolongation (T104I) and greatest average increase in triangulation (P197S) in TS2. **(C-E)** A selection of computational models from the population that showing more impact of the P197S mutation than the T104I mutation. **(F)** One computational model from the population responds to the T104I mutation with more AP prolongation than in response to the P197S mutation. (G-H) Example computational models from the population with similar response to both the T104I and P197S mutation.

Using the model optimization process shown in Fig 1 for TS1, we optimized the iPSC-CM model I_Ks_ to reflect kinetics and density measured from test set 2 (TS2) mutants. Only mutations with sufficient current density to measure V_1/2_ were included in the analysis. The optimized models of I_Ks_ for each mutation are shown in Fig 4A and 4B. A population of iPSC-CM models was made for each TS2 mutant, as described above for TS1.

Analyzing the cellular models within the TS2 mutant populations reveals profound phenotypic variability resulting from a given mutation. For example, individual cellular responses to two high severity LQTS mutations in TS2 are shown in Fig 4C–4H. The T104I and P197S mutations resulted in the largest mean APD_90_ prolongation (T104I) and the largest increase in AP triangulation (P197S). In comparing populations containing each of these mutants, we observed that some individual iPSC-CM models within the population were more sensitive to T104I (Fig 4C–4E), while a subset of different iPSC-CM models from the same population are profoundly sensitive to P197S (Fig 4F) and exhibit extensive cellular level disruption. Still, other example models are similarly impacted by both mutations (Fig 4G and 4H).

**Fig 5:**
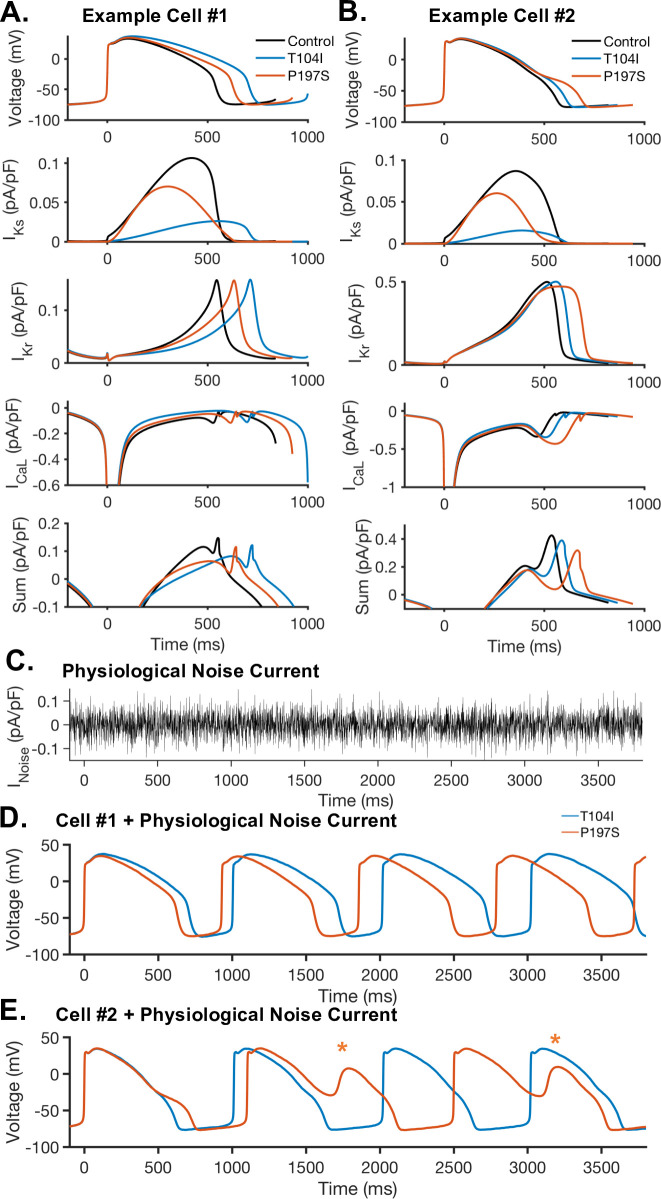
Comparison of ionic currents and response to physiological noise current in T104I and P197S mutant models. Sample action potentials from simulated cells with differential response to the T104I and P197S mutations are shown with their underlying currents. **(A)** Example cell #1 shows more AP prolongation in response to the T104I mutation. The underlying behavior of I_Kr_, I_Ks_, and I_CaL_ is shown during the AP, as well as the sum of these three currents (I_Kr_ + I_Ks_ + I_CaL_). **(B)** Example cell #2 shows more AP prolongation in response to the P197S mutation. The underlying behavior of I_Kr_, I_Ks_, and I_CaL_ is shown during the AP, as well as the sum of these three currents (I_Kr_ + I_Ks_ + I_CaL_). **(C)** Example of the physiological noise current applied to the cellular models to track beat-to-beat variability in response to noise. **(D)** Response of example cell #1 with physiological noise current. **(E)** Response of example cell #2 with physiological noise current. The application of low amplitude noise current reveals repolarizations abnormalities in the P197S mutation, as indicated with orange stars.

To further analyze mechanisms of differential response to the T104I and P197S mutations, we examined the underlying currents and response to a physiological noise current for the example models shown in Fig 4C and 4F. The ionic current behavior underlying the AP in each of these two cells are shown in Fig 5A and 5B. In example cell 1, the T104I mutation causes longer AP prolongation than P197S (Fig 5A). This difference in AP prolongation is driven primarily by the larger depletion in total I_Ks_ current caused by the T104I mutation. Example cell 1 exhibits similar mutant response when both mutations are modeled by G_Ks_ scaling alone (S1 Fig). Additionally, when the physiological noise current is applied (Fig 5C), P197S maintains a shorter APD than the same cell with the T104I mutation (Fig 5D). However, the opposite trend in AP prolongation is observed in example cell 2. In cell 2, P197S prolongs the AP more than T104I (Fig 5B). The shift in the time course of I_Ks_ during the AP caused by the P197S mutation results in an earlier peak I_Ks_, with near 0pA/pF I_Ks_ at the end of the AP (Fig 5B, orange, Time > 500ms). Example cell #2 model is more sensitive to the depletion in repolarizing current at the end of the AP, in part due to the balance of I_Kr_ and I_CaL_. Thus, the lack of I_Ks_ at the end of the AP in the P197S model results in the more severe AP prolongation for the P197S mutant compared to the T104I mutation. This is most clearly illustrated by the net current (I_Kr_ + I_Ks_ + I_CaL_) during the AP (Fig 5B, bottom panel), with the P197S mutation resulting in less net repolarizing current late in the AP. If the P197S and T104I mutants are modeled as G_Ks_ scaling alone (S1 Fig) the increased P197S prolongation is not observed. Thus, this increased AP prolongation due to P197S is captured due to the mutant model optimization to kinetic parameters (V_1/2_ and k). When the physiological noise current is applied, as shown in Fig 5E, the P197S mutation causes some APs to have repolarization abnormalities, as indicated by orange stars. As cell #2 is shown to be sensitive decreased new current late in the AP, the physiological noise current in the P197S mutant model is sufficient to prolong the AP further, and cause EADs.

### Test Set 2: Predicted LQTS Severity

**Fig 6:**
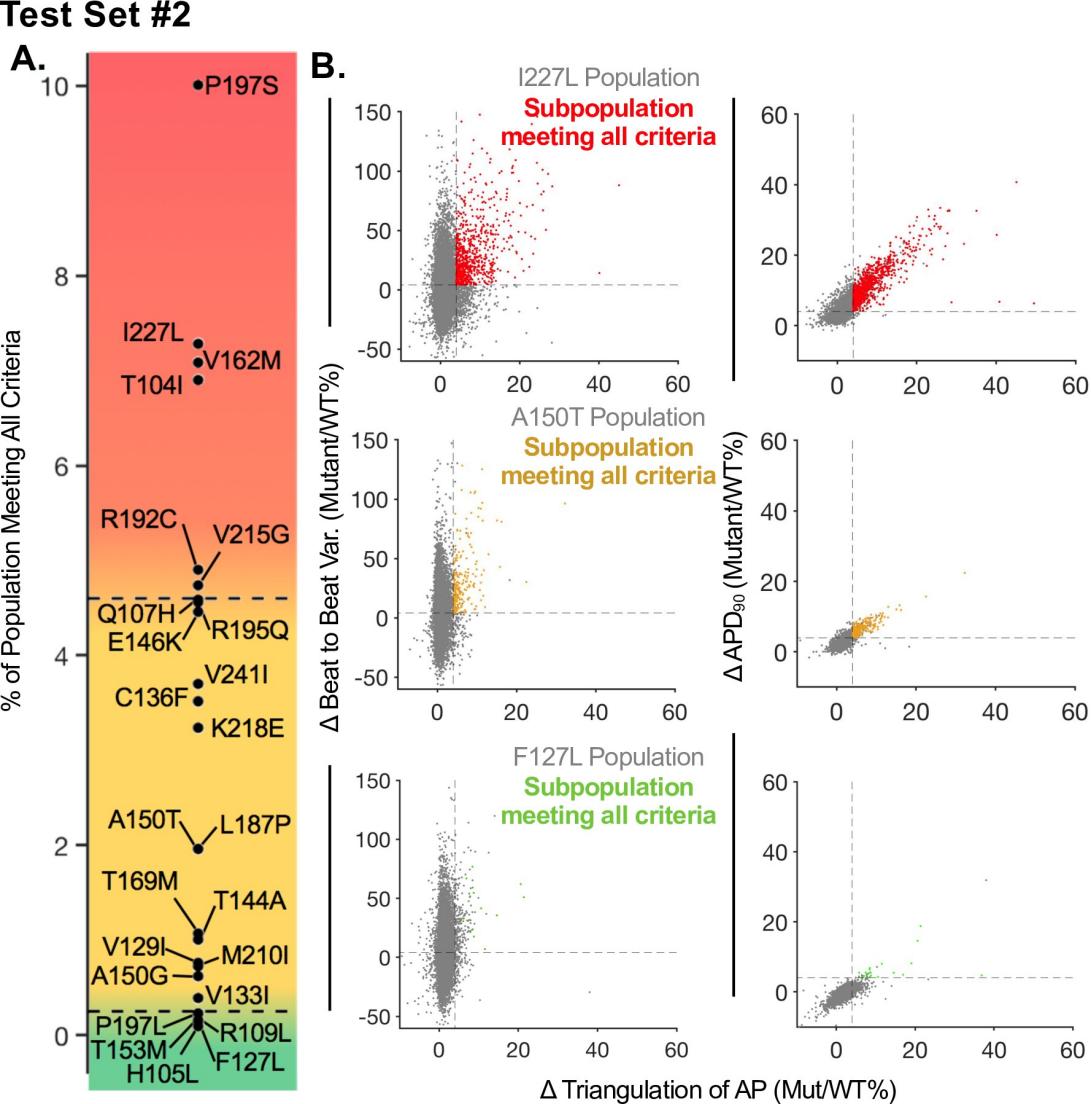
Prediction of LQTS severity in test set 2 using a population-based approach. The severity sorting framework was applied to test set 2 (TS2). TS2 includes mutations with unknown clinical phenotypes. **(A)** Predicted stratification of LQTS severity for TS2. Red corresponds to the most severe LQTS mutations, green corresponds to benign mutations for LQTS, and yellow highlights the region of severity which cannot be classified utilizing the framework developed with TS1. **(B)** Three example mutant populations showing the subpopulation of cells meeting the set criteria. Red highlighted points in the top row represent the 7.28% of cells in the I227L population which surpass the 4% threshold for all three criteria. Yellow highlighted points in the middle row represent the 1.96% of cells in the A150T population which surpass the 4% threshold for all three criteria. Green highlighted points in the bottom row represent the 0.16% of cells in the F127L population which surpass the 4% threshold for all three criteria.

Using the populations of iPSC-CMs models we predicted and classified the severity of each mutation in Test Set 2 (TS2) by applying the framework developed for TS1. The results of this analysis are shown in Fig 6A, where the color gradient indicates severity from high (red) to unclear phenotype (yellow) to mild (green). Fig 6B shows a more detailed analysis of the three exemplar mutations (one from each category, severe, unclear and mild LQTS risk) from TS2 where impacts were simulated in a population of iPSC-CM models. The categorization criteria (increase in beat-to-beat variability, APD_90_ prolongation, and increase in triangulation) as shown in Fig 6B.

The predicted severity of each mutation is compared to the Vanoye *et al*. predicted severity in Table 2. Vanoye *et al*. used the patch clamp acquired current density data for each KCNQ1 mutation to categorize severity of each mutation (Severe loss of function (LOF) mutations at <25% of WT current density, mild LOF 25–75% WT current density, etc.). Table 2 also shows the percentage of the wild-type model population excluded due to lack of spontaneous beating or repolarization failures for each mutant population. In general, severe mutations caused more repolarization abnormalities in the model population, resulting in a higher percentage.

**Table 2:** Comparison of Vanoye et al. and Computational Results for TS2.

Vanoye et al. Analysis		Computational Modeling Results			
Mutation	Current Density (%, exper.)	Mutation (Colored by Vanoye Classification)	Models meeting criteria (%)	Modeling Severity Prediction	Repolarization Failure (%)
P197S	0.17	P197S	9.94	Severe LQTS	6.1
R192C	0.21	I227L	7.22	Severe LQTS	4.4
T104I	0.22	V162M	7.05	Severe LQTS	4.0
I227L	0.22	T104I	6.83	Severe LQTS	4.4
C136F	0.23	R192C	4.86	Severe LQTS	4.5
V162M	0.24	V215G	4.73	Severe LQTS	3.9
V215G	0.33	Q107H	4.55		3.8
Q107H	0.36	R195Q	4.55		3.9
V241I	0.44	E146K	4.44		3.8
K218E	0.46	V241I	3.70		3.0
R195Q	0.49	C136F	3.51		4.4
T144A	0.5	K218E	3.21		3.7
A150T	0.58	A150T	1.93		3.6
M210I	0.63	L187P	1.95		3.5
E146K	0.65	T169M	1.06		3.4
T169M	0.65	T144A	1.00		3.4
V133I	0.67	V129I	0.74		3.3
T153M	0.67	M210I	0.72		3.4
L187P	0.68	A150G	0.62		4.1
F127L	0.8	V133I	0.36		3.6
V129I	0.83	P197L	0.23	Benign LQTS	12.3
A150G	0.93	R109L	0.23	Benign LQTS	11.1
H105L	1.31	T153M	0.14	Benign LQTS	3.0
P197L	1.47	F127L	0.16	Benign LQTS	5.3
R109L	2.23	H105L	0.09	Benign LQTS	6.3

SevereLOFMildLOF

NearNormalNormal

Severe GOF

Notably, GOF mutations (P197L, R109L, and H105L) also caused an increase in the number of models with repolarization failure. Characterization of the GOF mutations are shown in Table 3. GOF function mutation severity was examined using the same 4% threshold for beat-to-beat variation and triangulation criteria, as done for the LOF mutations. The criteria for APD_90_ was defined as the subpopulation of models with a 4% or more shortening of AP duration, compared to WT. However, without available clinical pathogenic characterization of GOF mutations, it is not possible to validate the predictions of pathogenic severity as was done for the comparison of TS1 and TS2 for LQT1.

**Table 3:** Computational Results for GOF mutations.

Mutation	Models meeting SQT criteria (%)	Repolarization Failure (%)
P197L	9.56	12.3
R109L	2.37	11.1
H105L	0.01	6.3

**Fig 7:**
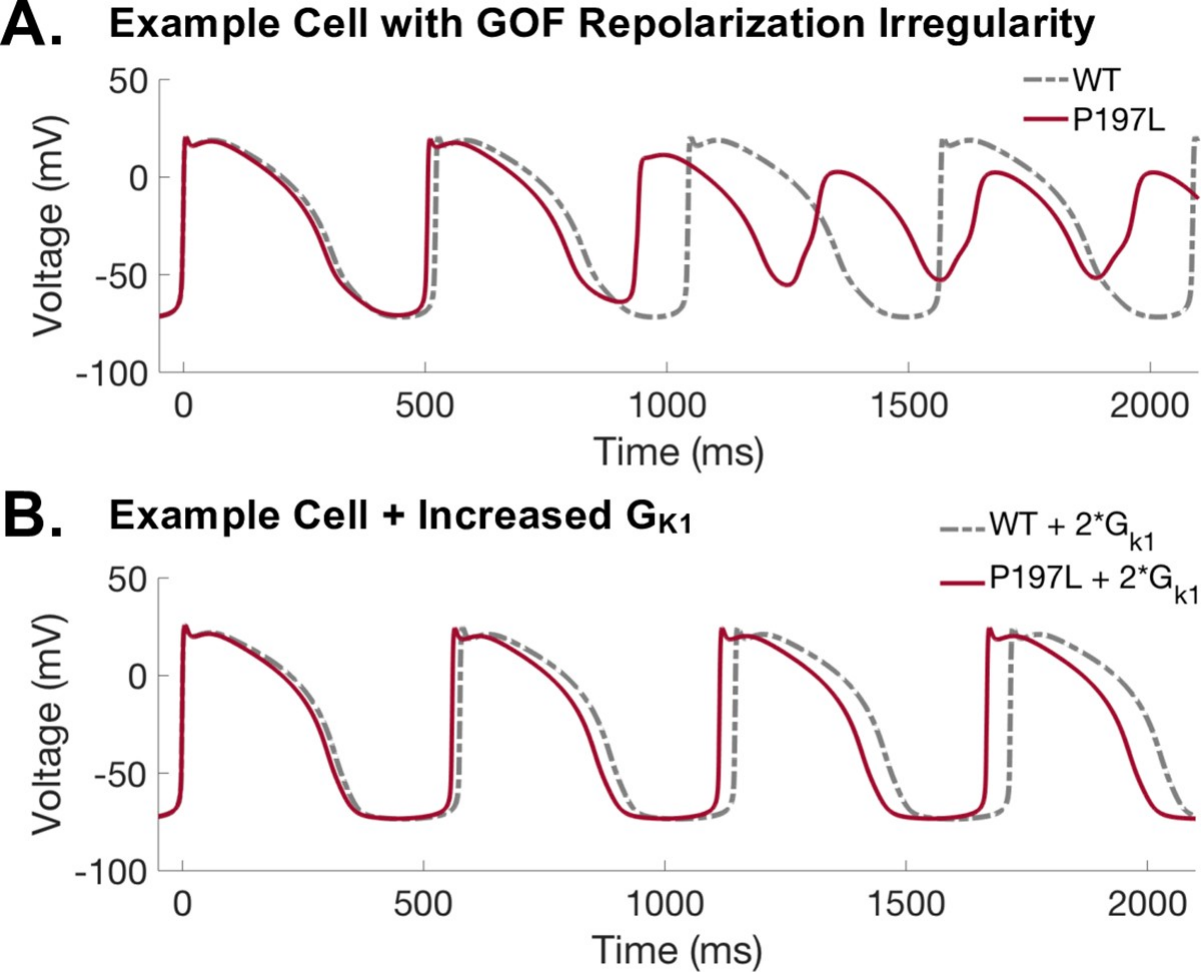
Example of repolarization irregularity in gain of function (GOF) mutant. **(A)** Sample action potential showing repolarization irregularity in P197L mutant, a GOF mutation, compared to wild-type. **(B)** Stabilization resting potential by increased in G_K1_. GOF mutant causes expected shortening of AP duration, and increase in G_K1_ prevents repolarization irregularity.

The increase in repolarization failure occurs in part because the increase in I_Ks_ due to GOF mutations can also cause increase in resting voltage of the AP. GOF mutations P197L, R109L, and H105L cause an average increase in maximum diastolic potential (MDP) of 0.5, 0.13, and 0.08 mV, respectively compared to WT. Severe LOF mutations I227L and V162M cause an average decrease in MDP of -0.13 and -0.16mV, respectively compared to WT. Due to spontaneous beating of all iPSC-CM models in the wild-type population, and the wide variability in our model populations, some model cells are highly sensitive to small changes in resting membrane voltage. An example of repolarization failure caused in part by instability in the resting membrane voltage is shown in Fig 7A. Repolarization failure was defined as failure to reach AP amplitude above 70 mV (see methods). Stabilizing the membrane voltage by simulating the same cell with an increase in I_K1_ can rescue the repolarization failure, as shown in Fig 7B. Increase in I_K1_ has also been shown to stabilize the iPSC-CM AP in several experimental studies [39–41].

Our model predictions are in good agreement with the reported data in the literature for the mutations in TS2 [42–49]. Interestingly, R195Q and Q107H were classified as mild LQTS mutations in the recent study by Vanoye et al, whereas our method (which utilized the Vanoye data) rather suggested a severe pathogenic phenotype, consistent with other *in silico* approaches [42, 43]. Additionally, while Vanoye *et al*. classified the T153M mutation as a mild loss of function, the cellular level iPSC-CM computational model method predicts T153M to be benign. In the simulated T153M mutant population, 0.14% model cells are above all three prediction criteria, and similarly our model predicts 0.09% model cells above all criteria for V207M mutant, a TS1 clinically benign mutant. This is consistent with the likely benign rationale discussed in the ClinVar entry for the T153M mutation [44]. Similarly, our approach predicted the C136F mutation to be less severe than the Vanoye *et al*. classification. Based on the distribution of the severity of mutations in TS1, C136F falls into the range of risk outputs which are not predicted by our framework as conclusively pathogenic or benign. This may be due to the fact that mutations within this range have incomplete penetrance or require additional environmental, pharmacological, or genetic perturbations to cause a pathogenic phenotype. This is consistent with the literature for five other mutations in TS2 which fall within this range (C136F [45], A150T [46], T144A [47], L187P [48], and K218E [49]).

It should be noted that the expression of these mutant channels is expected to vary from cell-to-cell and patient-to-patient. Furthermore, TS1 and TS2 variants were characterized by Vanoye et al. in a homozygous state, as opposed to being co-expressed with the WT channel *in vitro*. Within the homozygous state there is some experimentally observed variation parameters, including current density. This variability was not included in TS1 and TS2. S2 Fig (S2 Fig) shows example APs resulting from simulating variability in mutant maximal conductance. Example Cell 1 from Fig 5, is simulated using the base mutant model from TS2, compared to mutant models with variable current density within 2-times the experimentally observed standard error. While variation in the maximal conductance will cause some variation in disease phenotype, this variation is relatively small compared to the range of WT phenotypes included in the model population. To study the heterozygous state, test set mutants were expressed with the WT allele and characterized in Vanoye *et al*., as a model system for determining autosomal dominant traits. Based on the homozygous variant data, as discussed previously for TS1 and TS2, the selected mutants were all characterized as pathogenic in Vanoye *et al*. and our model system. Additionally, we have simulated model populations with I_Ks_ optimized to the experimental data collected from the WT/variant heteromultimeric channels, as shown in S3 Fig (S3 Fig.). Consistent with the Vanoye et al. results, the mutant phenotype was consistently less severe when the model is fit to the WT/variant data, rather than the variant/variant data as shown in Figs 4 & 6. However, only one mutant characterized in the WT/variant system has a known clinical pathogenic phenotype (G314S), and the majority of variants characterized have a similar impact on the model population, compared to the known pathogenic variant (S1 Fig.). Three mutants (I227L, V162M, G179A) seem to have a less severe whole-cell impact in the heterozygous system, consistent with the conclusions from Vanoye et al. As only one mutant with known clinical phenotype (G314S, pathogenic) was characterized in this heterozygous system, it is not presently possible compare a clinically know and unknown set of mutants, as was done for TS1 and TS2 in this study.

### Validation of KCNQ1 mutation effects in adult ventricular model

**Fig 8:**
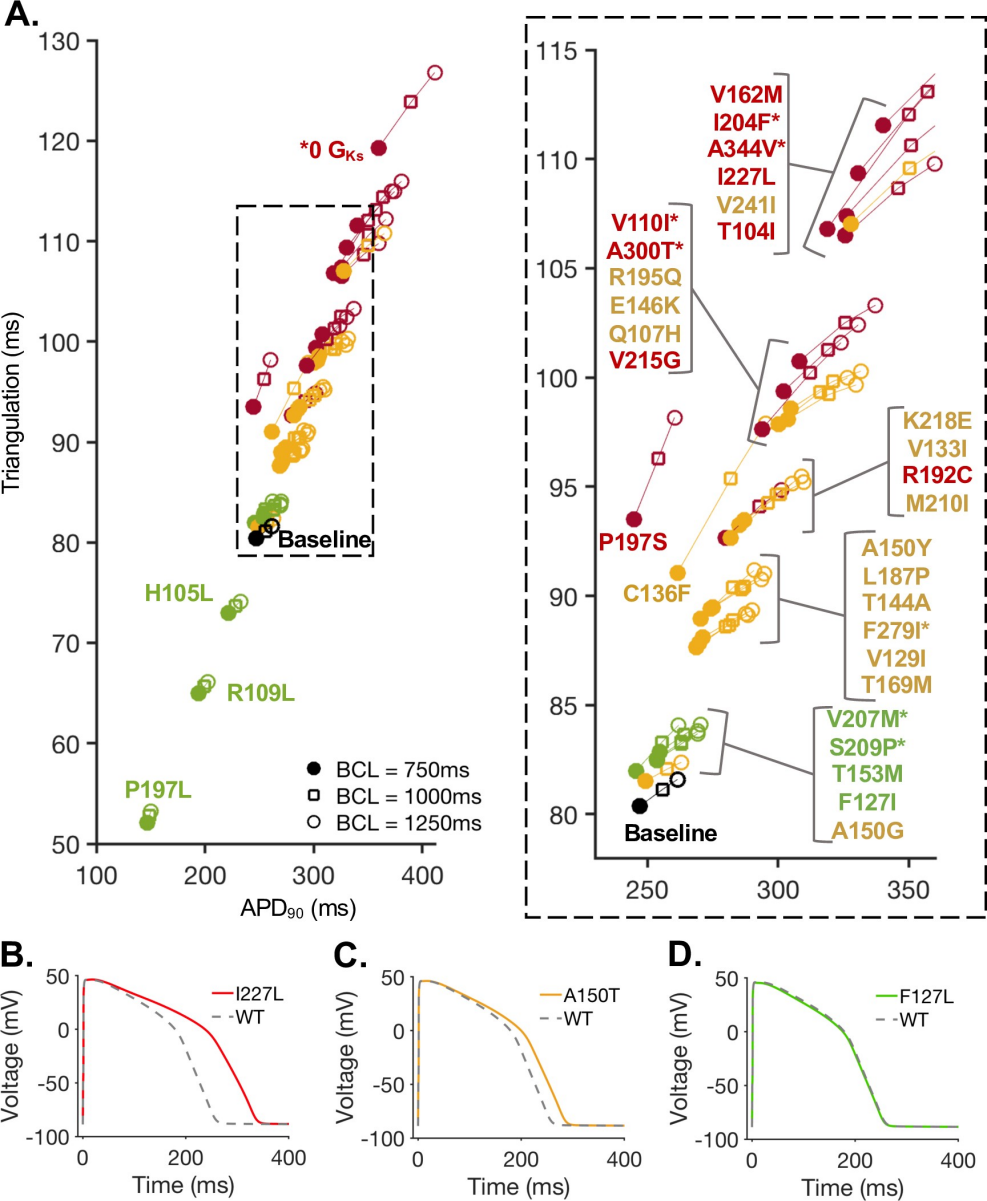
The predicted impact of I_Ks_ mutations from test set 1 and test set 2 in the O’Hara- Rudy computational model of the adult ventricular cardiomyocyte. **(A)** Each mutated I_Ks_ model was adapted for the adult ventricular cardiomyocyte model by incorporating relative changes in gating. APD_90_ and triangulation of the Mann et al. optimization of the O’Hara-Rudy computational model of the adult ventricular cardiomyocyte with each mutant I_Ks_ model is shown for three pacing rates with basic cycle lengths (BCL) or 750 ms, 1000 ms, and 1250 ms. Starred mutations (*) are from test set #1 (TS1), remaining mutations are from test set #2 (TS2). The color of each mutation is determined by the designated classifications in the iPSC-CM model populations, as shown for TS1 (Fig 3A) and TS2 (Fig 4A). **(B)** The adult ventricular cellular model AP for wild-type (grey, dashed) and I204F mutant (red) at cycle length (CL) 750 ms. **(C)** The adult ventricular cellular model AP for wild-type (grey, dashed) and A150T mutant (yellow) at CL = 750 ms. **(D)** The adult ventricular cellular model AP for wild-type (grey, dashed) and F127L mutant (green) at CL = 750ms.

In this study, we have examined the effects of a variety of KCNQ1 mutations in two test sets, TS1 and TS2 by predicted the impact of individual mutants in iPSC-CMs models. However, an important drawback of the iPSC-CM approach as both an experimental and simulated model system is that there is no clear way to determine how the model outputs will relate to adult phenotypic manifestation of genetic variants. For this reason, we next developed an adaptation for the computational models of KCNQ1 mutants that we tested in the iPSC-CMs models to allow for prediction of their effects on the adult cardiac ventricular myocyte (model formulation is described in the methods). Fig 8A shows the predicted relationship between APD_90_ and APD triangulation for each mutation in the Mann et al. optimization of the O’Hara-Rudy (ORd) adult ventricular cell model[50, 51]. The Mann et al. global optimization of the ORd model results in increased model APD prolongation is response to pathogenic LQT mutations, as compared to the original ORd Model. Interestingly, the mutations which were predicted to result in the most severe phenotypes in the iPSC-CM modeling approach also show the largest increase in AP duration and triangulation in the adult ventricular cell model. The mutation effects on the AP are shown for example mutations in Fig 8B–8D, and the trend across all 33 mutations analyzed is in consistent agreement with the predictions from the iPSC-CM population.

## Discussion

Induced pluripotent stem-cell derived cardiomyocytes (iPSC-CMs) are a promising reagent utilized in a variety of methods to reveal human cardiac behavior in a physiological context and expand understanding of cardiac disease and drug response. Currently, the use of iPSC-CMs is limited by three main factors: (1) difficulty in representing phenotypic variability experimentally, (2) low throughput electrophysiological measurements, and (3) the immature phenotype may not accurately represent adult cardiac behavior in all conditions. In this study we developed a computational modeling and simulation approach to address all three limitations and serve as a complementary approach to *in vitro* studies.

We utilized experimental data describing KCNQ1 kinetics and current density in CHO cells to develop computer models of normal and mutant cardiac slowly activating delayed rectifier potassium current I_Ks_. Through development of these computational models, the impact of each mutation can be observed within the cellular context, in order to reveal the mutation effect on whole-cell behavior. The wild-type and mutant current models were incorporated into populations of iPSC-CM computer models that we recently developed and described [31]. By making predictions across a population of computer models representing the observed range of cell-to-cell variation in iPSC-CM electrophysiology, we were able to predict the impact of mutations on a computer-based representation of diverse genetic backgrounds and thus observe rare events. The framework allowed for an efficient mode of analysis for many the range of emergent behaviors arising from mutations. The range of observed behaviors indicate that population modeling has some advantages over previously developed approaches which focus on average cellular level impacts of mutations [52].

Experimental data describing functional impacts of mutations with ***known*** clinical phenotypes were labeled test set 1 (TS1). Data from this group of mutations was used to develop a computational framework for assessing LQTS severity. By applying random parameter variation from within experimentally reported data ranges to develop a population of iPSC-CM models [31], we predicted a wide range of phenotypic responses to each mutation.

We stratified the TS1 iPSC-CM mutant model populations by severity through tracking the fraction of model cells that exhibited diseased phenotypes. We tracked multiple parameters including AP prolongation, AP triangulation, and beat-to-beat variability. The model correctly separated mutations that were observed clinically to be pathogenic or benign. Using these results and the clinically observed outcomes for the TS1 mutants, we determined pathogenic “cut-offs” and then applied them to predict the severity of mutations in test set 2 (TS2). We also assessed the impact of all mutants in the adult setting by simulating their effects in the O’Hara-Rudy adult cardiac ventricular myocyte model. It was notable that the predicted impacts of individual mutations in the immature iPSC-CM mutant model populations were consistent with the predictions that emerged in the adult model cell populations, suggesting that the iPSC-CM model system may indeed provide valuable and relevant genotype phenotype information.

Comparing the predictions from experimental data in Vanoye *et al*., where the authors utilized ionic current as the severity indicator and our computational model framework that relies on whole-cell membrane potential predictions (Table 2), reveals some differences arising from the two approaches. While Vanoye *et al*. classified the T153M mutation as a mild loss of function, the cellular level iPSC-CM computational model method predicts T153M to be benign. It should be noted that the Stanford Center of Inherited Cardiovascular disease submission in ClinVar for this disease categorizes the mutation as likely benign due to its common minor allele frequency (MAF) in the general population, but a lack of confirmed LQTS diagnosis [44]. The consistent prediction of the computer model with the ClinVar database may indicate the importance of a population-based approach where emergent cellular level phenotype predictions suggest that most genetic backgrounds mask the impact of the mutation.

The Q107H and R195Q mutations have previously been studied in mechanistic studies, which predicted the mutations to be likely pathogenic in alignment with our prediction of these mutants near the pathogenic range. For Q107H, a structural modeling study concluded that the mutation would destabilize folding, suggesting a disease mechanism [42]. Additionally, the KCNQ1 mutation R195Q was evaluated by Clemens et al. and showed that 6 of 8 *in silico* phenotyping algorithms utilizing genetic sequence information classified the R195Q mutation as pathogenic [43]. The iPSC-CM model population, by accounting for the impact of other ionic currents, compensatory behavior in the whole-cell, and variability in response between patients and cells, allows for a more specific interpretation of risk (and associated score) for mutations between the severe and benign classifications. The predictions suggest that both Q107H and R195Q fall near the border of severe and mild LQT severity scores (Fig 6) and may be pathogenic for a fraction of patients dependent on the underlying genotype. The cellular level predictions seemed to even account for incomplete penetrance in a population, which is consistent with recessive A300T mutations from TS1 [36], and resulted in a similar LQTS severity score (A300T = 4.8, Q107H = 4.6, R195Q = 4.6).

There are additional instances where the cellular level iPSC-CM computational modeling and simulation approach did not agree with the Vanoye *et al*. classification. An example is the C136F mutation. Based on the distribution of the severity of mutations in TS1, C136F falls into the range of risk outputs which are not predicted by our framework as conclusively pathogenic or benign. The range of mild mutations (yellow, Table 2), has an interesting clinical prevalence in the literature. Three mutations (C136F [45], A150T [46], and T144A [47]) have each respectively been identified in a single patient diagnosed with LQTS using genotyping of LQTS patients. However, existence of a mutation in a single patient is insufficient information to conclude pathogenicity. Although a single mutation in an LQTS-susceptible gene in a patient exhibiting prolonged QTc may indicate causality, other interpretations include; (1) the patient has other contributing factors, including genetics, making them especially sensitive to a mutation, or (2) the patient has a completely separate genetic mutation causing the disease phenotype.

The alternative gene cause is exemplified in the case of another mutation in TS2: V133I. The V133I mutation, which our method predicts to be near benign, was identified in a patient case of sudden cardiac death (SCD). However, it was subsequently discovered that the KCNQ1 V133I was unlikely to be the cause of the SCD, and it was instead caused by a separate pathogenic desmin contractile protein mutation [53]. This serves as an example where low-throughput clinical observation in a single patient is insufficient to predict pathogenicity. However, in combination with the methods presented in this study, we can suggest that a mutation is sufficient to cause disease or suggest the likelihood of an alternative explanation for a given phenotype.

Two other TS2 mutations (L187P and K218E) which fall within the predicted mild phenotype range (yellow, table 2 and Fig 6A) suggest that these mutations may require additional contributing factors to cause pathogenicity. Zhang *et al*. studied the L187P mutation in a family with some members showing QTc prolongation, but 58% of family members possessing the L187P mutation had normal to borderline prolonged QTc [48]. This suggests a mild clinical penetrance of the L187P mutation, which resulted in a VUS classification in ClinVar. The incomplete penetrance of the L187P mutations may be due to additional gene modifiers, environmental factors, or other patient-to-patient variations which protect against or promote the LQT phenotype in some family members. Prior studies have shown that LQT mutations often show incomplete penetrance [54, 55], and additional gene modifiers can amplify potentially pathogenic mutants [56–59].

Another example of mutations that depend on additional perturbations to cause LQTS are acquired, or drug-induced, Long QT (aLQTS) mutations, where mutations are only associated with disease phenotypes in the presence of drugs. In TS2, the K218E mutant was associated with the development of arrhythmias in the presence of dofetilide [49]. Genetic mutations linked to drug-induced QT prolongation have been observed for many LQT related genes [60–64], and the mechanisms of these aLQTS mutations has been explored *in silico* [65, 66]. The iPSC-CM mutant model framework can be readily expanded to include genetic and drug impacts in future studies.

The evidence shows mutations such as L187P and K218E, which are classified as mild in the iPSC-CM mutant model predictions, can cause LQTS with concomitant genetic predisposition or additional perturbations. It is possible that other mutations with a similar predicted outcome (V241I, C136F, A150T, T144A, and T169M) require additional perturbations to exhibit pathogenic phenotypes. Due to the broad parameter space we sampled in the iPSC-CM computational model population, we did identify some instances that are particularly susceptible to LQT and may be representative of naturally occurring genotypes which possess predisposition to LQT [67]. Patients with multiple mutations in LQTS genes have increased risk of life-threatening cardiac events, so it is also possible that a single mutation is safe for most patients but in combination with additional mutations becomes life threatening [68].

There are several limitations of this study which may impact the severity of the characterized mutations. The experimental data used to characterize these I_Ks_ models was conducted in a homozygous system, despite that LQTS is often inherited in an autosomal dominant manner. As experimental characterization of the complete TS1 and TS2 mutations were not been conducted in the heterozygous state (co-expression of mutant and WT KCNQ1), It is also not possible to know if in humans, the heterozygous state results in 50% transmission of mutant and WT alleles, as multiple other states are possible including dominant negative effects and mosaicism. Vanoye et al. did include an analysis of the most severe homomeric channel phenotypes in the WT/mutant heteromultimeric channels, as modeled in Supplemental S3 Fig (S3 Fig.). Furthermore, even in the homozygous state, there can be variability in the maximal current density, as observed in Vanoye et al. and simulated in S2 Fig (S2 Fig.). Many other factors which may also impact the severity of these mutations, including activation of the sympathetic nervous system. Since sympathetic nervous systems has been shown to increase the severity of these mutations, we would anticipate an increase in severity of mutation. However, as we have only made predictions for TS2 relative to TS2, and both test sets were conducted experimentally under the same conditions. In the future, this computational modeling approach could be expanded to include contributions from these pathways, as has been done in other models of cardiomyocytes[69, 70].

Finally, the prediction of the mutation effects in the adult model system represents a first step toward utilizing phenotypic variability in iPSC-CM modeling to make predictions in the adult system. In the near future, deep learning based “translation” approaches to convert iPSC-CM modeling results to adult cardiomyocyte response can be used to expand the methodology presented in this study. The analysis of LQT1 mutants in the adult model serves a validation of the utility of modeling mutations in the iPSC-CM system, while utilizing the phenotypic variability incorporated in the iPSC-CM model system to examine cell-to-cell variability in response to mutations. An ideal system would model iPSC-CM mutations, use the iPSC-CM model system to extrapolate population-based iPSC-CM response to mutations, and translate the iPSC-CM population to an adult patient-population of models. Utilizing the currently available experimental data and iPSC-CM modeling approaches, this study is a first step toward applying iPSC-CM phenotypic variability to understand variation in disease expression.

The modeling approach applied in this study serves as an *in silico* complement to existing methods to linking genotype to phenotype. We have utilized experimental and clinical data to develop I_Ks_ mutant models and determine the range of pathogenic and benign behaviors in the model framework. Together this allowed for the simulation of the impact of LQT1 mutations and VUS on diverse phenotypes and predict the severity of mutations without known clinical outcomes. Finally, by using a computational model to simulate the impact of mutations from both the immature iPSC-CM models to the O’Hara-Rudy adult ventricular cardiac myocyte model, we have demonstrated the impact of mutations across the continuum of aging. In the future, the approach can be expanded to examine additional cardiac mutations or pharmacological interventions. Furthermore, a population-based approach will be critical in identifying patient phenotypes that are particularly susceptible to unintended drug effects and developing multi-drug treatments to mitigate those effects.

## Methods

### IKs Model Optimization

As described in our previous study of an iPSC-CM model [31], I_Ks_ gating was modeled using single-exponential rate functions such that:

$$I_{Ks}=G_{Ks}*x_{act}^{2}*(V_{m}-E_{K})$$ Eq (1)

$$x_{act,\infty }=\frac{1}{1+{\frac{x_{3}}{x_{1}}e}^{V(\frac{1}{x_{4}}-\frac{1}{x_{2}})}}=\frac{1}{1+{x_{6}e}^{V{*x}_{7}}}$$ Eq (2)

$$\tau_{x,act}=\frac{1}{{x_{1}e}^{V/x_{2}}+{x_{3}e}^{V/x_{4}}}+x_{5}=(\frac{1}{{x_{1}e}^{V{*x}_{2}}}*x_{act,\infty })+x_{5}$$ Eq (3)

Model parameters x_1-5_ and G_Ks_ were optimized for each mutation simulated. I_Ks_ mutants were optimized based on the shift in V_1/2_ and k (slope) of steady-state activation (x_act,∞_), and the current density (G_Ks_). Experimental data reporting the change in each parameter between cells expressing the wild-type (WT) and mutated KCNQ1 channel, as reported in Vanoye et al, were used for model optimization. For example, for the I204F mutation Vanoye *et al*. reported a 14.8 mV positive shift in V_1/2_ between the control and mutant experimentally measured activation curves. Thus, our I204F I_Ks_ model was fit to a 14.8 mV shift in V_1/2_ from our previously published WT iPSC-CM I_Ks_ model. Similarly, the changes in k and current density compared to WT, as measured experimentally in Vanoye *et al*., was used to further constrain the steady-state activation curve and determine G_Ks_, respectively.

### Defining Test Sets of Mutations

All mutations modeled were characterized experimentally in Vanoye *et al*. [30]. The experimental study included a training set of mutations and a test set of mutations, which served as the basis of the two sets of mutations modeled in this study: test set 1 (TS1) and test set 2 (TS2). TS1 includes the 15/30 mutations in the Vanoye et al. training set with known clinical phenotypes in the ClinVar database [33]. Of these mutations 8/15 mutations were modeled as complete I_Ks_ block (G_Ks_ = 0), due to insufficient current to characterize these mutations experimentally.

TS2 includes all mutations in the Vanoye *et al*. test set (25/48 mutants) which had sufficient experimental data to optimize the I_Ks_ model (V_1/2_, k, and current density). The remaining 23 mutations included in the Vanoye et al. test set had insufficient current to characterize a model, and would be modeled as complete I_Ks_ block, as done in TS1. For clarity we did not include Vanoye et al. test set mutations which resulted in complete I_Ks_ block (E115G, G119R, Y125D, H126L, L131P, L134P, S140R, F167del, R174H, R174L, W176R, G186C, G189A, G189E, R190P, R195P, D202G, Q234P, L236P, L236R, G245R, G179A, and S225W). Mutations in TS2 have unknown or conflicting clinical assessments in the ClinVar database.

### Implementation of Model Populations

To analyze the impact of individual mutants on whole cell electrical behavior in the simulated iPSC-CM, we incorporated each I_Ks_ mutation in a population of iPSC-CM models developed previously in Kernik *et al*. [31]. We utilized the model population which included variation in five major ionic currents (I_Na_, I_CaL_, I_Kr_, I_K1_, and I_f_). In Kernik *et al*. (2019), variation was modeled by fitting the model to multiple experimental kinetic datasets to predict phenotypic variability in the electrical response of the whole-cell model. This model population can serve to represent phenotypic variability in response to LQT mutations. The published control model population did not include variation in the I_Ks_ model parameters. Here, we applied to perturbations to the I_Ks_ parameters to replicate the effect of each mutation and then tested the impact of the I_Ks_ mutant in the population. For example, for the I204F mutant, every cell in the simulated iPSC-CM population contains the six parameters that were optimized to the experimental data for I204F.

The simulated iPSC-CM population comprised 11422 model cells described in Kernik *et al*. [31]. All “wild-type” cells were deemed to have met the inclusion criteria if they were spontaneously beating and fully repolarizing (AP amplitude over 70 mV, resting voltage below −40mV, no alternans, and no repolarization abnormalities)_._ Each I_Ks_ mutation was then incorporated into each cell in the population and the simulation was allowed to run until steady-state was achieved. Steady state was defined by a <1% change in minimum ion concentrations between the first and last beat of a 50s simulation run. Steady-state conditions were the same as defined for the control iPSC-CM population in Kernik *et al*. After reaching steady-state (ranging from 60-600s), a 20s simulation was run starting at steady-state initial conditions, and AP morphology markers were analyzed. Additional AP properties were then analyzed during simulation with physiological noise, as described in the next section. Severity indication analysis was conducted using the model subpopulation that retained normal repolarization with the mutation based on the inclusion criteria: AP amplitude over 70 mV, no alternans, and no repolarization abnormalities. This was done in part to account for the impact of the elevated resting potential within the model population, as observed in iPSC-CMs.

Instability of the resting voltage and spontaneous beating due to a lack of I_K1_ is a critical consideration when utilizing iPSC-CMs as a model cell-type [39]. Due to the lack of I_K1_, and resulting elevated resting potential in some iPSC-CMs, a fraction of LQT1 mutant iPSC-CM model cells do not produce a complete AP after loss of repolarizing current in LQT. These “incomplete” APs were identified as APs with amplitude <70mV. Similar abnormalities are seen experimentally, where injected I_K1_ was required to observe drug-induced APD prolongation in some iPSC-CMs [41, 71], or APD prolonging drugs were observed to stop spontaneous beating in iPSC-CMs [72]. However, cessation of spontaneous beating is not inherently a proarrhythmic quality of cardiac cells, as normal adult ventricular cardiomyocytes do not beat spontaneously [73]. To remain consistent between our previous analysis of the wild-type iPSC-CM model population and to allow comparison of mutant and wild-type outputs in each cellular model, models with repolarization abnormalities were not included in our severity analysis. However, models which developed repolarization failures in the mutant population were tracked, and the percentage of models with repolarization failures are listed in Tables 1 and 2.

### Physiological Noise Current

Each model cell was subject to a physiological noise current application, with noise applied after steady state was reached. Each cell was simulated for 40 beats with physiological noise current applied, and the final beat was saved for analysis. Simulations for test set 1 were undertaken for variable duration (20, 30, and 40 beats with physiological noise). The duration of the simulation did not impact the results. The noise current (I_Noise_) was formulated as additive Gaussian white noise of amplitude 0.045 pA/pF. This amplitude was determined by matching the amplitude of I_Noise_ based on prior calculations of physiological noise current in cardiac myocytes [74]. For each simulation with I_Noise_, beat-to-beat variability and triangulation were analyzed. Beat-to-beat variability was defined as the absolute value of APD_90,n_-APD_n90,n+1_ averaged for beat n = 1 to beat n = 39. Triangulation of each beat is calculated as APD_90_-APD_30_ for each action potential.

### Adult Ventricular Models

The O’Hara-Rudy (ORd) ventricular model, as optimized by Mann *et al*. for LQT, was used to predict adult response to mutants, referred to as the Mann-ORd model [50, 51]. From the original ORd model, the Mann-ORd model used in this study includes scaling factors for G_Ks_, G_CaL_, the sodium-calcium exchanger (I_NCX_), and the sodium-potassium pump (I_NaK_), optimized in Mann *et al*. to recapitulate the LQT phenotype in adult ventricular cardiomyocytes. G_Ks_ from the iPSC-CM WT model was scaled by a factor of 3.5 to adapt the iPSC-CM I_Ks_ model to the Mann-ORd whole-cell model. This scaling factor was determined by replacing the ORd I_Ks_ model with the iPSC-CM I_Ks_ WT model, and scaling G_Ks_ to maintain APD_90_ from the baseline Mann-ORd model (APD_90_ = 248 ms at BCL = 750 ms) For each mutant I_Ks_ model, the same G_Ks_ scaling factor was used to convert the iPSC-CM I_Ks_ mutant model to the adult mutant I_Ks_ model. All other parameters from the iPSC-CM I_Ks_ mutant models were retained in the adapted adult mutant models.

## Supporting information

S1 Fig Comparison of ionic currents in T104I and P197S mutants modeled with G_Ks_ scaling only. Sample action potentials from simulated cells with T104I and P197S mutations modeled by only scaling G_Ks._ G_Ks_ scaling factors were the WT-normalized Vanoye et al. current density measurements (Table 2). Example cell WT models are the same as in Fig 5 **(A)** Example cell #1 shows more AP prolongation in response to the T104I mutation, similar to mutant response shown in Fig 5. The underlying behavior of I_Kr_, I_Ks_, and I_CaL_ is shown during the AP, as well as the sum of these three currents (I_Kr_ + I_Ks_ + I_CaL_). **(B)** Example cell #2 shows nearly identical prolongation in response to T104I and P197S mutations, unlike the model with mutant I_Ks_ kinetics. With mutant kinetics, as shown in Fig 5, there was more prolongation with the P197S mutant. The underlying behavior of I_Kr_, I_Ks_, and I_CaL_ is shown during the AP, as well as the sum of these three currents (I_Kr_ + I_Ks_ + I_CaL_).
(TIF)

S2 Fig Simulated APs for WT example cell 1 (as shown in Fig 5) compared with the base mutant model (as simulated for TS2, shown in Fig 5), and mutant models with ±4% and ±8% change in GKs scaling factor. G_Ks_ is scaled to 16%, 20%, 24%, 28% and 32% of WT G_Ks_ for the -8%, -4%, base mutant, +4%, and +8% simulated traces, respectively.
(TIF)

S3 Fig The severity sorting framework was applied to KCNQ1 mutants characterized in the WT/variant heteromultimeric channel. The G314S mutant is the only included mutant characterized as clinically pathogenic, and is highlighted in red. **(A)** Predicted stratification of LQTS severity based on percentage of population meeting all three criteria for each mutant. **(B)** Table summarizing simulated results for each mutant. Models meeting criteria is the same as shown in panel A. Repolarization failure is determined as done for TS1 and TS2 in Tables 1–3.
(TIF)
