# Supplementary figures and images for: A computational model of induced pluripotent stem-cell derived cardiomyocytes for high throughput risk stratification of KCNQ1 genetic variants
Source: PLoS Comput Biol. 2020 Aug 14;16(8):e1008109. doi: 10.1371/journal.pcbi.1008109 (PMC7449496; doi:10.1371/journal.pcbi.1008109)

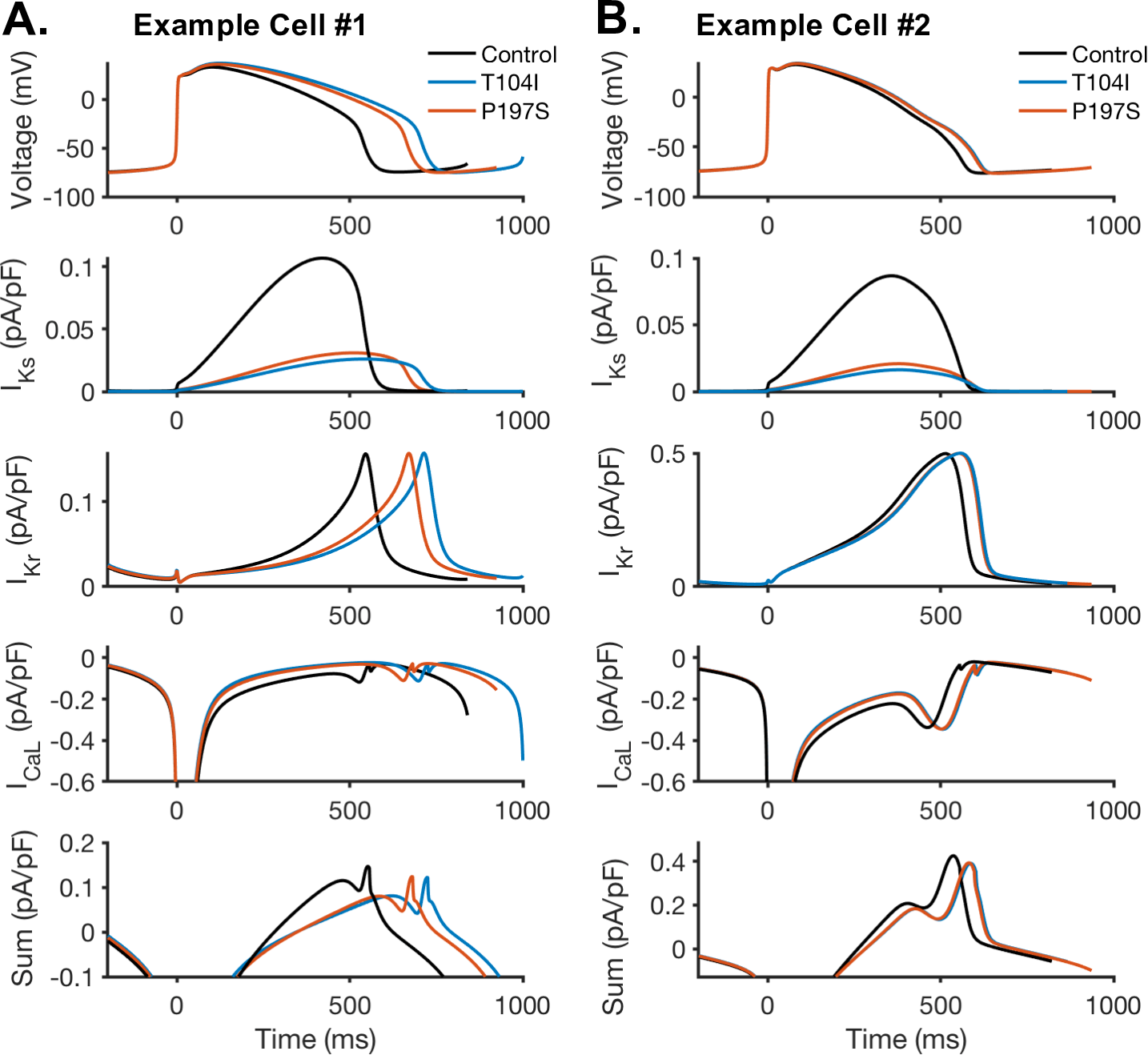

Supplement: S1 Fig — Sample action potentials from simulated cells with T104I and P197S mutations modeled by only scaling GKs. GKs scaling factors were the WT-normalized Vanoye et al. current density measurements (Table 2). Example cell WT models are the same as in Fig 5 (A) Example cell #1 shows more AP prolongation in response to the T104I mutation, similar to mutant response shown in Fig 5. The underlying behavior of IKr, IKs, and ICaL is shown during the AP, as well as the sum of these three currents (IKr + IKs + ICaL). (B) Example cell #2 shows nearly identical prolongation in response to T104I and P197S mutations, unlike the model with mutant IKs kinetics. With mutant kinetics, as shown in Fig 5, there was more prolongation with the P197S mutant. The underlying behavior of IKr, IKs, and ICaL is shown during the AP, as well as the sum of these three currents (IKr + IKs + ICaL). (TIF) [file pcbi.1008109.s001.tif]

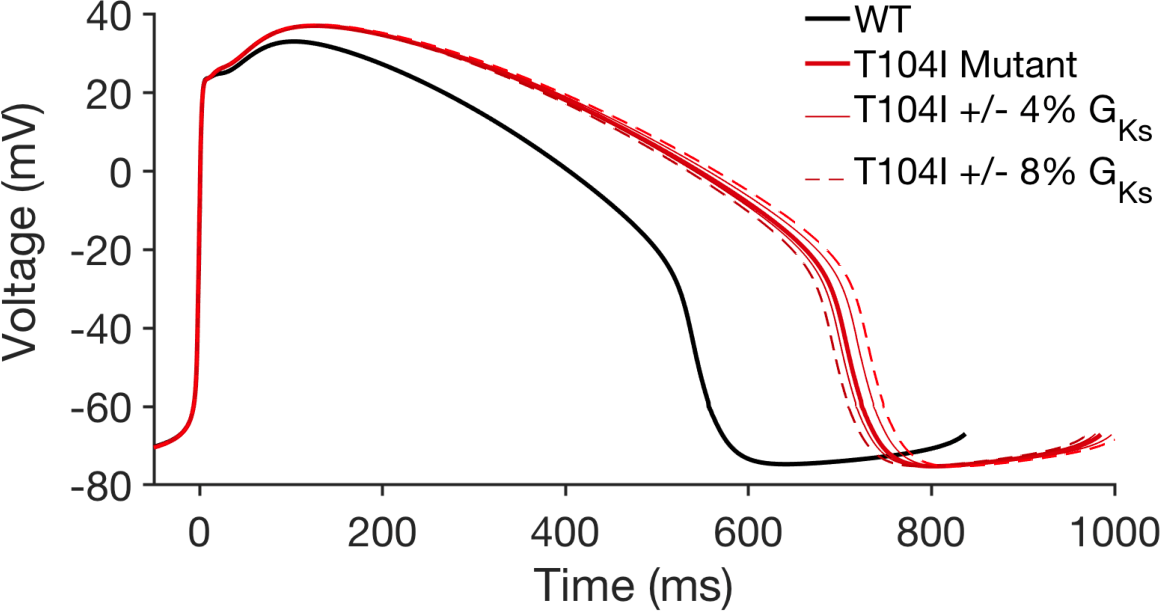

Supplement: S2 Fig — GKs is scaled to 16%, 20%, 24%, 28% and 32% of WT GKs for the -8%, -4%, base mutant, +4%, and +8% simulated traces, respectively. (TIF) [file pcbi.1008109.s002.tif]

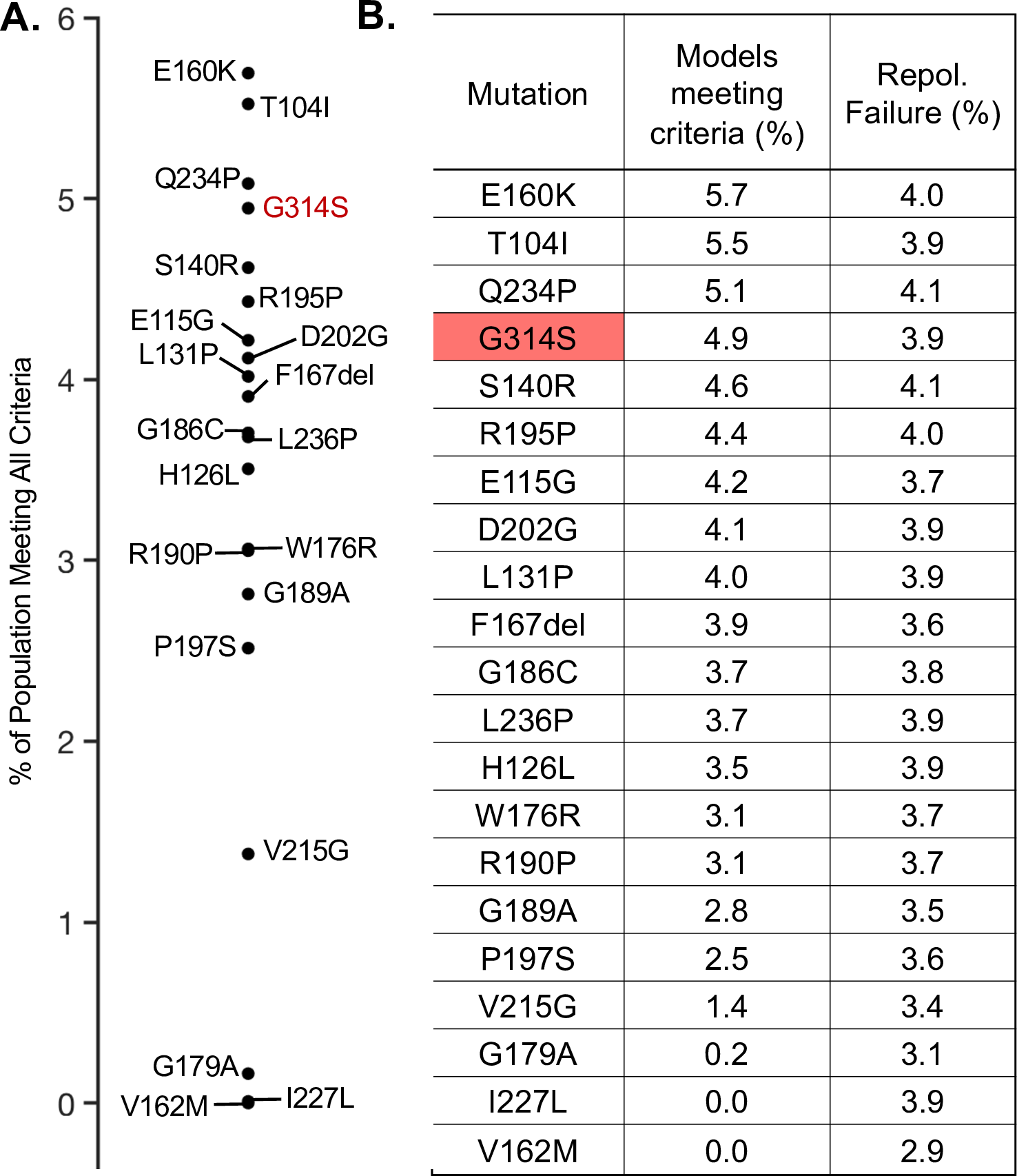

Supplement: S3 Fig — The G314S mutant is the only included mutant characterized as clinically pathogenic, and is highlighted in red. (A) Predicted stratification of LQTS severity based on percentage of population meeting all three criteria for each mutant. (B) Table summarizing simulated results for each mutant. Models meeting criteria is the same as shown in panel A. Repolarization failure is determined as done for TS1 and TS2 in Tables 1–3. (TIF) [file pcbi.1008109.s003.tif]
